# The Tp0684 (MglB-2) Lipoprotein of *Treponema pallidum*: A Glucose-Binding Protein with Divergent Topology

**DOI:** 10.1371/journal.pone.0161022

**Published:** 2016-08-18

**Authors:** Chad A. Brautigam, Ranjit K. Deka, Wei Z. Liu, Michael V. Norgard

**Affiliations:** 1 Department of Biophysics, The University of Texas Southwestern Medical Center, Dallas, TX, 75390, United States of America; 2 Department of Microbiology, The University of Texas Southwestern Medical Center, Dallas, TX, 75390, United States of America; Consiglio Nazionale delle Ricerche, ITALY

## Abstract

*Treponema pallidum*, the bacterium that causes syphilis, is an obligate human parasite. As such, it must acquire energy, in the form of carbon sources, from the host. There is ample evidence that the principal source of energy for this spirochete is D-glucose acquired from its environment, likely via an ABC transporter. Further, there is genetic evidence of a D-glucose chemotaxis system in *T*. *pallidum*. Both of these processes may be dependent on a single lipidated chemoreceptor: Tp0684, also called TpMglB-2 for its sequence homology to MglB of *Escherichia coli*. To broaden our understanding of this potentially vital protein, we determined a 2.05-Å X-ray crystal structure of a soluble form of the recombinant protein. Like its namesake, TpMglB-2 adopts a bilobed fold that is similar to that of the ligand-binding proteins (LBPs) of other ABC transporters. However, the protein has an unusual, circularly permuted topology. This feature prompted a series of biophysical studies that examined whether the protein’s topological distinctiveness affected its putative chemoreceptor functions. Differential scanning fluorimetry and isothermal titration calorimetry were used to confirm that the protein bound D-glucose in a cleft between its two lobes. Additionally, analytical ultracentrifugation was employed to reveal that D-glucose binding is accompanied by a significant conformational change. TpMglB-2 thus appears to be fully functional *in vitro*, and given the probable central importance of the protein to *T*. *pallidum*’s physiology, our results have implications for the viability and pathogenicity of this obligate human pathogen.

## Introduction

The sexually transmitted disease syphilis is re-emerging as a global health threat [1–8], motivating continued research on the disease’s spirochetal etiologic agent, *Treponema pallidum*. Although this obligate human pathogen is a diderm like Gram-negative bacteria, the cell envelope has unique features. Particularly notable are the scarcity of outer-membrane proteins [9–13] and the fact that periplasmic proteins tend to be membrane-anchored via fatty-acyl groups post-translationally added to their respective processed N-termini [14]. This class of proteolipids, also known as “lipoproteins”, are involved in many vital cellular processes, including nutrient import and flavin homeostasis [15–22]. Because *T*. *pallidum* cannot be cultivated *in vitro*, a structure-to-function approach of this organism’s cadre of lipoproteins has provided new insights into its biology and, more generally, molecular microbiology [15–29].

The acquisition of nutrients, such as carbon sources, nitrogen sources, and cofactors, is a major undertaking for most microorganisms. For *T*. *pallidum*, these needs are particularly pressing. The bacterium has a minimized genome [30] of only 1,041 genes; in comparison, *Escherichia coli* strain K-12 has over 4,400 [31]. Thus, *T*. *pallidum* lacks the machinery for the *de novo* synthesis of many key nutrients, including nucleotides, riboflavin, fatty acids, and amino acids [30]. It must therefore devote considerable resources toward acquiring these nutrients from its human host. In many cases, this is accomplished via multi-protein ABC transporters. In prototypical Gram-negative bacteria, the ligand-binding protein (LBP) components of these transporters exist free in the periplasm, but in *T*. *pallidum*, they are often lipoproteins that are likely anchored to the outer leaflet of the cytoplasmic membrane. Characterizations of these LBPs have revealed in *T*. *pallidum* likely transporters for transition metals [16,19], purine nucleosides [15], methionine [17], riboflavin [20], and polyamines [18]. Many others probably are present, but verifications of their probable functions typically are impeded by the need for heterologous hyper-expression in *E*. *coli* and *in vitro* characterization of recombinant molecules.

All non-photosynthetic organisms must acquire energy sources from their external milieu. For *T*. *pallidum*, the preferred energy source likely is glucose [32–35], as this monosaccharide is more abundant in human plasma than alternative carbon sources [36], and the spirochete contains all of the necessary enzymes for glycolysis [30]. Further, the organism probably has a means for the conversion of reducing equivalents from glycolysis to electrochemical potentials that can be utilized for further ATP generation [28]. The acquisition of glucose, therefore, is of paramount importance to this human pathogen. Bioinformatic analysis of the open reading frames in the genome of *T*. *pallidum* reveals two possible lipoproteins that could be the ligand-binding components of a glucose ABC-type transporters. They are Tp0545 and Tp0684 [30,37]. Because of their sequence homologies to *E*. *coli* MglB, they have been termed TpMglB-1 and TpMglB-2, respectively. In *E*. *coli*, MglB has a dual function: it serves as the periplasmic ligand-binding protein of an ABC-type transporter, and it is also the chemoreceptor for galactose chemotaxis [38]. Earlier studies on the soluble portion of TpMglB-2 indicated that it binds both glucose and galactose with nearly equal facility [37].

To further elucidate the function(s) of TpMglB-2, we heterologously hyper-expressed the soluble domain of the protein and characterized it structurally and biophysically. The 2.05-Å crystal structure of TpMglB-2 revealed a fold that is similar to other glucose/galactose binding proteins. However, the topology of this fold differed from those of LBP exemplars. Bioinformatic studies showed that this topology is in putative glucose/galactose-binding proteins in other treponemes and is also present in proteins from many species of Gram-positive bacteria. This divergent topology prompted us to perform more finite scrutiny of the binding and hydrodynamic characteristics of the protein. Using isothermal titration calorimetry (ITC) and differential scanning fluorimetry (DSF), we found that the protein exhibited a marked preference for binding to D-glucose over D-galactose, and that no other assayed carbon source bound detectably to the protein. Analytical ultracentrifugation (AUC) studies were consistent with TpMglB-2 being a monomeric protein that changes its conformation upon binding its preferred substrate, D-glucose. When these studies are coupled with genetic considerations, TpMglB-2 appears to be the founding member of a new topological class of LBPs of ABC-type glucose transporters.

## Results

### The crystal structure of TpMglB-2

Purified, recombinant TpMglB-2 readily formed crystals that diffracted X-rays to a minimum Bragg spacing of 2.05 Å (Table 1). The determination of the structure was accomplished using single-wavelength anomalous diffraction data from a selenomethionyl derivative of the protein (see Materials & Methods). The final model after refinement featured excellent geometry, and 366 of the 387 residues present in the construct were visible in the electron-density maps. The missing residues were mostly in the N-terminal tag, with only two internal residues (L26 and T27) not visible. Note that throughout this report we utilize a numbering scheme based on the mature form of the native TpMglB-2, counting the N-terminal cysteine residue of the mature protein as residue 1.

**Table 1:** Data collection, phasing, and refinement statistics.

Data Set	TpMglB-2	SeTpMglB-2
PDB Accession No.	5JX2	
**Data Collection**		
Space Group	C222_1_	C222_1_
Unit Cell Dimensions (Å)		
a	77.556	76.095
b	130.025	109.927
c	88.250	89.549
α = β = γ (°)	90	90
Resolution (Å)	36.78–2.05 (2.09–2.05)^a^	46.8–2.0^b^ (2.03–2.0)
Completeness (%)	100.0 (100.0)	99.9 (99.4)
Multiplicity	5.6 (5.6)	6.4 (3.6)
Unique Reflections	28,333 (1,373)	25,584 (1,253)
*R*_*merge*_^c^	0.075 (0.604)	0.138 (0.550)
<*I*>/*σ*_*I*_	19.6 (2.9)	12.7 (2.6)
Wilson B (Å^2^)	30.8	26.4
**Phasing**		
Sites Found	N/A	10
Overall Figure of Merit	N/A	0.296
Automatically Built Residues	N/A	342
**Refinement**		
Resolution (Å)	36.78–2.05	
No. Non-Solvent Atoms	2,791	
No. Solvent Atoms	191	
Maximum-Likelihood Coordinate Error (Å)	0.20	
**Average *B*-factors**		
Non-Solvent (Å^2^)	43.95	
Solvent (Å^2^)	43.21	
***R*-values**		
*R*_*work*_^d^	0.186	
*R*_*free*_^e^	0.226	
**Ramachandran Statistics**		
Outliers (%)	0.3	
Most Favored Region (%)	96.7	
**r.m.s. deviations**		
Bonds (Å)	0.004	
Angles (°)	0.842	

^a^Numbers in the parentheses are reported for the highest-resolution shell of reflections.

^b^Only data to 2.2 Å resolution were used for phasing purposes.

^c^ $R_{merge}=\sum_{hkl}\sum_{i}|I_{h,i}-\left\langle I_{h}\rangle \right|/\sum_{hkl}\sum_{i}I_{h,i}$ where the outer sum (*hkl*) is over the unique reflections and the inner sum (i) is over the set of independent observations of each unique reflection.

^d^ $R_{work}=\sum_{hkl}\left|\left|F_{o}\right|-\left|F_{c}\right|\right|/\sum_{hkl}\left|F_{o}\right|$, where *F*_*o*_ and *F*_*c*_ are observed and calculated structure factor amplitudes, respectively.

^e^*R*_*free*_ is calculated using the same formula as *R*_*work*_, but the set *hkl* is a randomly selected subset (5%) of the total structure factors that are never used in refinement.

**Fig 1:**
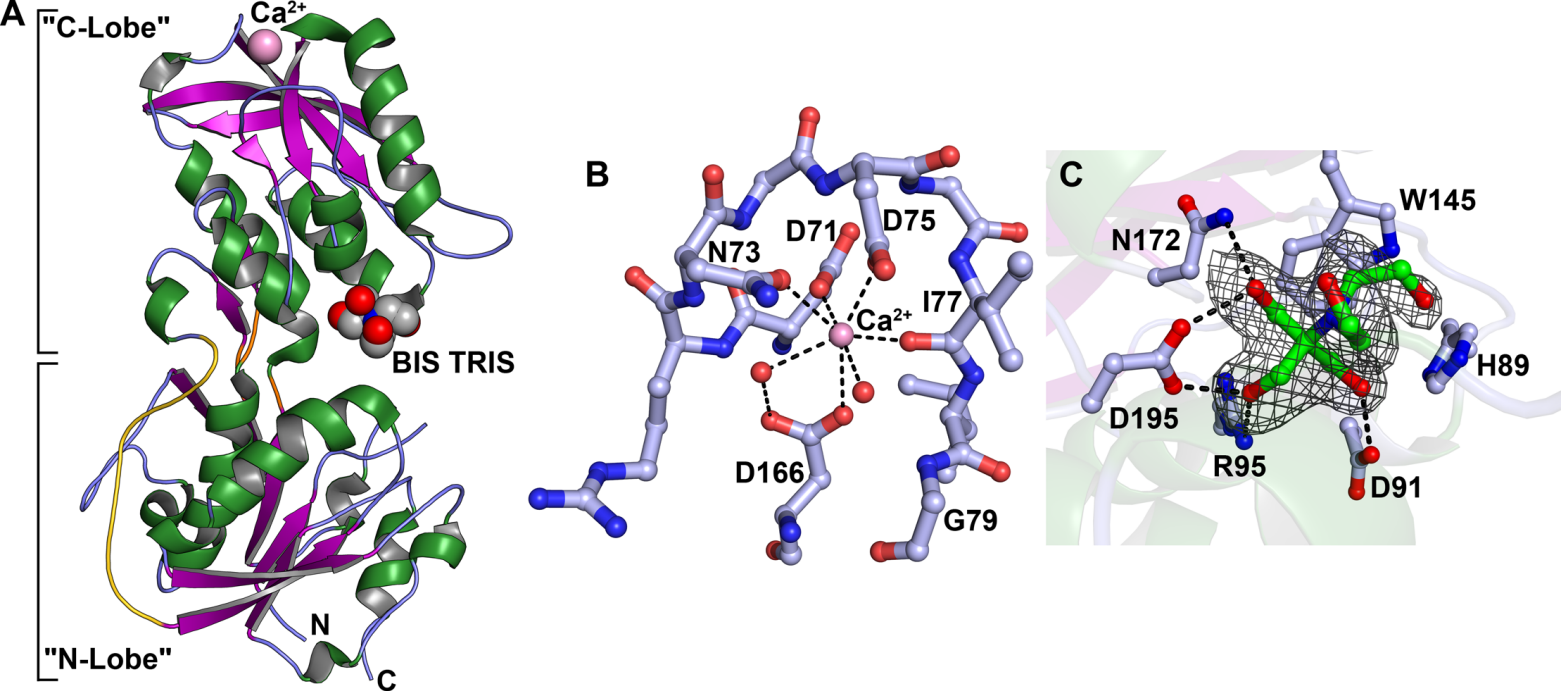
Structural aspects of TpMglB-2. (A) The overall structure. Shown in ribbons representation is the overall crystal structure of TpMglB-2. Helices (either α- or 3_10_) are shown in green, β-strands are purple arrows, and regions without regular secondary structure are light blue. The three canonical hinge regions are colored orange. The adventitiously bound ligand BIS-TRIS is shown as a group of spheres between the protein’s two lobes. Oxygen atoms are colored red, nitrogen atoms blue, and carbon atoms are gray. The Ca^2+^ ion bound to the C Lobe is shown as a pink sphere. The span of the protein that forms the fourth hinge region and also comprises the connecting motif not observed in EcMglB (see text) is colored gold. (B) The Ca^2+^-binding site. Residues 71–79 are shown, with carbon atoms in light blue, and other atoms colored as described in (A). Water molecules are shown as red spheres. Inner-sphere contacts to the Ca^2+^ ion are depicted as black dashes. (C) The BIS-TRIS binding site. A kicked omit *mF*_*o*_-*DF*_*c*_ map [43,44] is shown contoured at the 3-*σ* level and superposed on the refined coordinates of BIS-TRIS. Hydrogen bonds between the protein and the buffer molecule are drawn as black dashes. H89 is shown because it is in van der Waals contact with the molecule. Its ε N atom is about 3.3 Å from the proximal hydroxyl group on the BIS-TRIS, but this distance coupled with the poor geometry make it unlikely that these two atoms are hydrogen-bonded.

The overall fold of TpMglB-2 (Fig 1) resembles those of LBPs (sometimes called “Periplasmic Binding Proteins”) that serve as receptors for nutrients and cofactors in bacterial ABC transporters [39,40]. LBPs generally have two structurally similar lobes that are attached by a hinge region. When ligand is absent, there usually is a large, solvent-exposed cleft between the lobes, and the protein displays an open, extended conformation. However, when the cognate ligand binds in the cleft, the two lobes clamp down on it, resulting in a more closed, compact, conformation. This mechanism has been compared to a “Venus fly trap” [41]. In a structural homolog (see below) of TpMglB-2, *E*. *coli* MglB (EcMglB) [42], these lobes were termed the “N” and “C” lobes because these domains harbored the N- and C-termini of the protein, respectively. Although this rationale fails for TpMglB-2 (both N- and C-termini are found in the same lobe), we choose to retain this nomenclature herein. Hence, the N lobe of TpMglB-2 contains the first visible amino-acid residue, R11, and the C lobe is located on the opposite side of the protein from this terminus (Fig 1A). There are four regions of the macromolecule that cross over between the two domains, forming the hinge region of the protein. The large cleft region is solvent-accessible, and thus this structure of TpMglB-2 appears to be in the “open” conformation.

The N lobe is dominated by a central, twisted β-sheet comprising seven β-strands. All but one of the strands are parallel. This sheet is almost completely surrounded by α-helices, 3_10_ helices, and other parts of the protein that do not adopt a regular secondary structure. The C lobe is organized similarly, but the central β-sheet has only six strands; again, one of them is antiparallel to the others. Decorating one edge of the sheet are two β-strands that have a strong curvature, giving the impression of a partial β-barrel. These strands are not counted as belonging to the central β-sheet, inasmuch as one of their surfaces is completely exposed to solvent.

Two small molecules are found in contact with the C lobe. The first is a calcium ion, which is located at the far end of the lobe, about 30 Å distant from the cleft (Fig 1A). This cation is coordinated by atoms from four side chains of TpMglB-2: D71, N73, D75, and D166 (Fig 1B). Other liganding interactions are provided by the main-chain oxygen atom of I77 and two water molecules. The Ca^2+^ appears to stabilize a loop on the C lobe while simultaneously adhering it to the main body of the domain. It thus likely plays a structural role, ensuring the proper folding and integrity of the C lobe.

The second small molecule is located in the interlobe cleft in association with residues of the C lobe; it is BIS-TRIS, which had been included in both the crystallization medium and the cryoprotection buffer at a concentration of 100 mM (see Materials & Methods). This molecule is stacked on the indole group of W145 (Fig 1C). Hydrogen bonds to hydroxyl groups on the buffer molecule are provided by the side chains of residues D91, R95, N172, and D195, and an additional van der Waals interaction is made by the side chain of H89. Although this site likely overlaps with the ligand-binding site (see below), the fact that TpMglB-2 adopts an open conformation with this small molecule bound suggests that its binding is adventitious.

### Comparisons of TpMglB-2 to other LBPs

Hidden-Markov-model-based searches [45] of the Protein Data Bank for sequences similar to that of TpMglB-2 revealed a large number of highly probable matches (over 100 matches with a greater than 99.9% probability of being a true positive). Most of these matches were LBPs from ABC transporters, and of these, most were annotated as sugar-binding proteins or putative sugar-binding proteins. Significantly, none of the matches had significant sequence homology to the final 60–100 amino acids of TpMglB-2.

**Fig 2:**
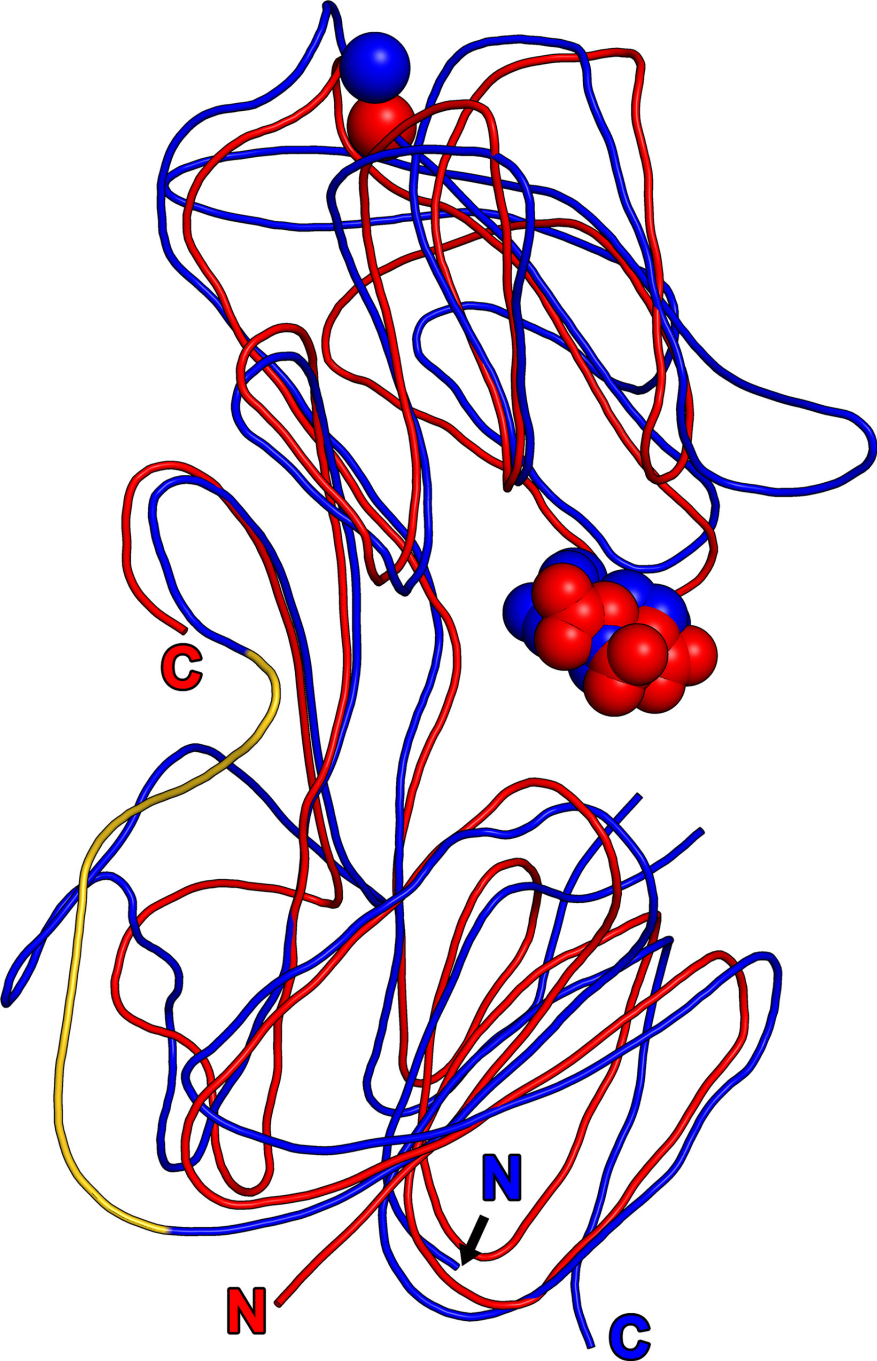
Superposition of TpMglB-2 and EcMglB. For both structures, a smoothed trace through the main chain is drawn. TpMglB-2 is shown in blue, while EcMglB is red. The bound ligands are depicted as spheres and are colored according to their respective protein chains, and the respective N- and C-termini are noted, with the letters color-coded to the respective structures. The gold coloration for the fourth hinge/connection motif from Fig 1 is retained here.

Secondary-structure matching [46] was used to search the Protein Data Bank for structures similar to that of TpMglB-2. Only two matches were located with substantial coverage and root-mean-square deviations (r.m.s.d.’s) on C_α_ positions of less than 3 Å. These matches were two different structures of *E*. *coli* MglB (EcMglB), in agreement with predictions that these structures would be similar [47]. In both of the matching structures, the EcMglB was in an “open” conformation. The best match (2.2 Å r.m.s.d. over 276 aligned C_α_ atoms) was to an apo form of EcMglB [48]. The other matching structure (2.3 Å r.m.s.d. over 277 aligned C_α_ atoms) was in an open conformation because a non-native ligand, 3-*O*-methyl glucose (3-OMe Glc), was bound to D-glucose-binding residues in the C lobe, apparently sterically precluding closure of the cleft [49]. Strikingly, when TpMglB-2 and 3-OMe-Glc-bound EcMglB were superposed using the proteins only, the two non-native ligands were in similar positions (Fig 2). Because the 3-OMe Glc and native glucose-binding sites overlapped, this implies that BIS-TRIS occupies the ligand-binding site of TpMglB-2 in the present crystal structure (Fig 1). Furthermore, this alignment allowed us to confirm that nearly all of the glucose-binding residues in EcMglB were identical in TpMglB-2 (Table 2). The only changes in the identities of these residues were conservative: D14 and N256 in EcMglB were analogous to N311 and Q215, respectively, in TpMglB2.

**Table 2:** Glucose-binding residues from EcMglB and their equivalents in TpMglB-2 and TpMglB-1.

EcMglB	TpMglB-2	TpMglB-1
D14	N311	N18
F16	F313	L20
N91	N19	N93
H152	H89	H154
D154	D91	S156
R158	R95	C160
W183	W145	D185
N211	N172	N212
D236	D195	D241
N256	Q215	N261

Notably, both proteins also possess Ca^2+^-binding sites in analogous positions (Fig 2). In EcMglB, the similarity of the arrangement and identity of amino-acid side chains liganding the calcium has been likened to the EF-loop of EF-hand proteins [42]. This likeness is generally preserved in TpMglB-2, with some differences. In EcMglB, the calcium cation is liganded by seven oxygen atoms from the protein, but in TpMglB-2, only five of the seven ligands emanate from the protein (Fig 1B). The remaining two coordinating atoms are oxygen atoms from water molecules. One of these differences arises from the fact that the coordinating side chain Q142 in EcMglB is not present in TpMglB-2; its equivalent is the non-liganding G79. Another difference is caused by the fact that D166 in TpMglB-2 does not make a bidentate contact to the Ca^2+^, as its EcMglB analog, E205, does. The consequences of these deviations from the usual EF-loop are unclear; no function (other than structural stabilization) has been posited for the Ca^2+^-binding site in EcMglB, as metal binding does not affect sugar binding [50].

**Fig 3:**
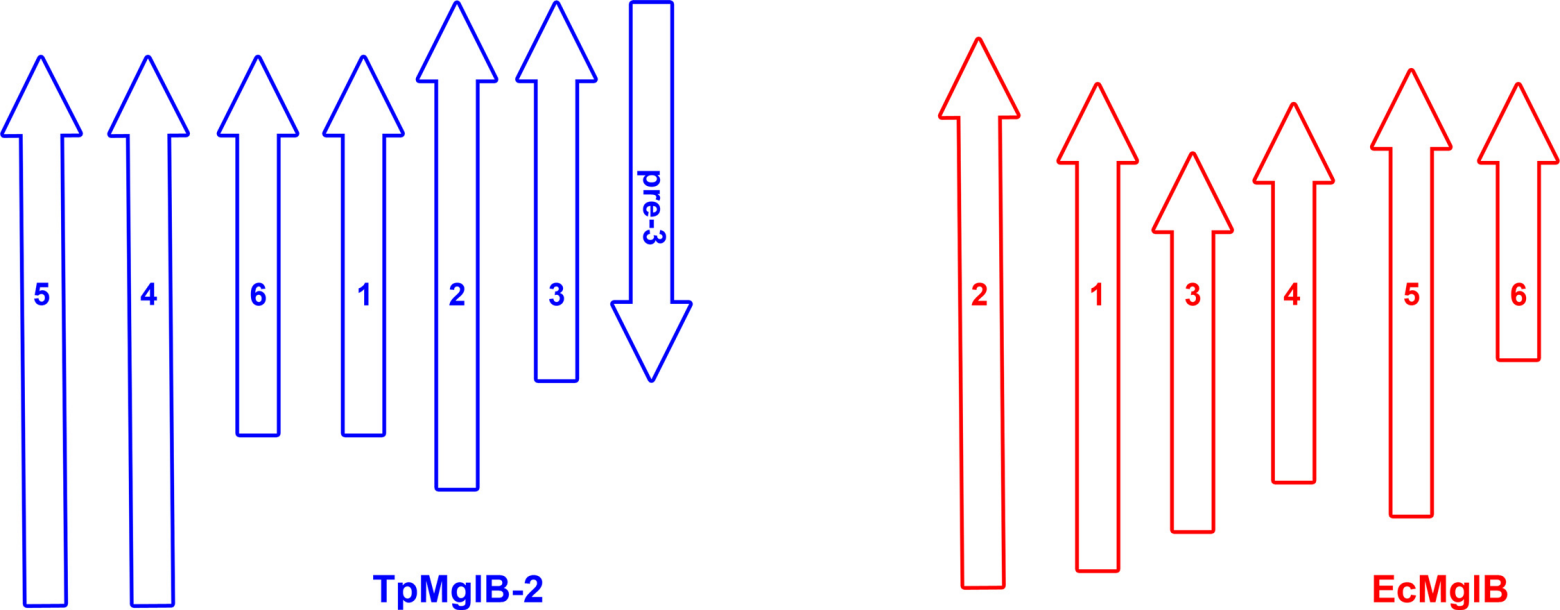
Comparison of the topologies of the central β-sheets in the N-lobes of TpMglB-2 and EcMglB. The β-strands for the respective sheets are drawn as arrows, with those from TpMglB-2 shown in blue and those from EcMglB in red. The strands are numbered according to the orders in which they appear in the primary structures of their respective proteins. The positions and sizes of the strands are approximately to scale. The antiparallel strand in TpMglB-2 was not assigned its own number in this scheme because it does not correspond to any strand in EcMglB. Its designation, “pre-3”, indicates that it immediately precedes strand 3 in the primary structure of TpMglB-2.

Comparisons of the structures of TpMglB-2 and EcMglB also reveal a stark difference in the proteins: their topologies are circularly permuted. This is conveniently illustrated via a comparison of the topologies of the central β-sheets of respective N lobes (Fig 3). If the strands of these sheets are renumbered according to their order in the respective proteins’ sequences, the order of the strands (from left to right in Fig 3) in EcMglB is 2-1-3-4-5-6. In two related classification systems for LBPs, the ordering of the first five strands is typical for a Type I [39] or “Cluster B” [40] LBP. However, the strand ordering in TpMglB-2 is 5-4-6-1-2-3. Hence, the first three strands in EcMglB are equivalent to the last three in TpMglB-2. Viewing this arrangement more globally, the first three strands in this lobe in EcMglB are near to the protein’s N-terminus (residues 3–9, 34–40, and 62–65), whereas the equivalent strands are close to the C-terminus of TpMglB-2 (residues 300–307, 331–337, and 358–361). The orders of the strands for the respective C lobes are unaffected by the permutation, and they adhere to the canonical 2-1-3-4-5 arrangement for Type I/Cluster B LBPs.

This reordering of the N lobes has additional consequences in the hinge region. The hinges of Type I/Cluster B LBPs are usually made up of three regions of irregular secondary structure representing points where the primary structure crosses over from one lobe to the other. The permutation noted above in TpMglB-2 necessitates a fourth cross-over; this region of the protein (residues 284–288) is close to the canonical hinge. It leads to an extended structure that spans from this fourth hinge region to the beginning of the permuted strand 4 described above. This fourth hinge and the following extended structure (colored gold in Figs 1 & 2) are not present in EcMglB.

The discovery of this permutation explains why the sequence searches could not match the final 70–100 residues of TpMglB-2. That is, this sequence should not be homologous to the C-termini of MglB homologues. If there is any sequence homology to the C-terminal portion of TpMglB-2, it should be to the N-termini of EcMglB and its homologues. An attempt to align the last 100 residues of TpMglB-2 and to the first 100 residues of EcMglB yields a poor match with only 13% sequence identity. For comparison, the last 230 amino acids of EcMglB can be aligned with the first 280 residues of TpMglB-2, featuring 26% identities.

**Fig 4:**
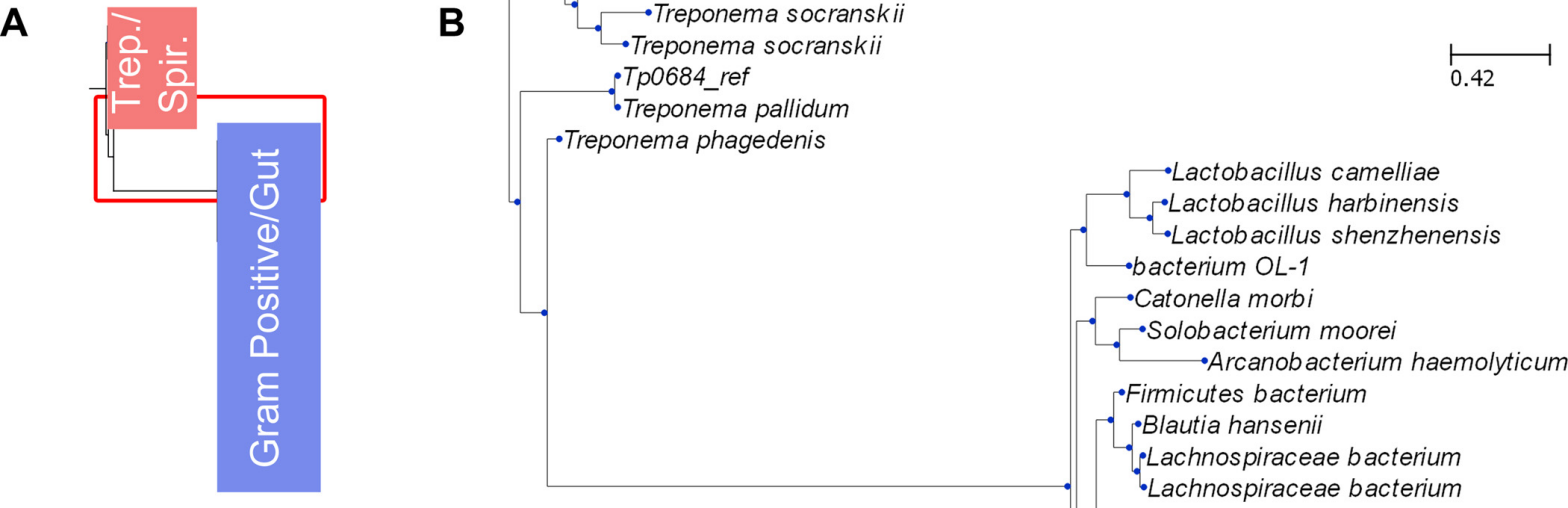
Relationships between proteins containing sequence and topology homology to TpMglB-2. (A) A schematic representation of the full cladogram. There are two main clades, which are shown as colored rectangles. The pink “Trep./Spir.” rectangle is occupied by treponemal and spirochetal organisms, while the “Gram Positive/Gut” blue rectangle is inhabited mainly by gram-positive organisms, many of which are mammalian gut commensal organisms. The red rectangle details the area of the figure that is blown up in (B). The full cladogram is available as a text file in the data supplied in S1 File. (B) A close-up of a branch point in the cladogram. The area of the cladogram highlighted in red in (A) is shown. TpMglB-2 is represented twice here (“Tp0684_ref” and “*T*. *pallidum*”) because it was included both as a structural template (in.pdb form) and as its native sequence. There is a slight difference between the two because of the 2 missing amino acids in the.pdb file.

These structural differences between TpMglB-2 and EcMglB prompted us to search sequence databases for proteins with both homologous sequences and similar topologies to that of the treponemal protein. This was accomplished using BLAST [51] and setting as a criterion that the coverage of the match be > 90% and that the N-termini matched well. A large number of matches were discovered. The top 100 matches were pooled, and, after winnowing identical or very similar sequences, a phylogeny was constructed (Fig 4; we included the sequence of EcMglB and that of the glucose-binding protein from *Salmonella typhimurium* as an outgroup).

The proteins with similar topologies to TpMglB-2 fall into two main groups: those from organisms of the phylum *Spirochaetes* and those largely from Gram-positive bacteria. While the former are mostly from treponemes, the latter are a mixture of mammalian pathogens and commensal bacteria. We also searched for sequence homologues to the gene product of *tp0686*, which is the putative permease for the TpMglB-2’s cognate transporter. The distribution of homologs was similar to that of TpMglB-2, with close homologs appearing in other treponemes and slightly more distant ones found in Gram-positive bacteria. The distribution of these proteins in a disparate group of human parasites, spirochetes, and commensal organisms suggests the possibility that these genes have been shared horizontally among these organisms or their ancestors.

The evident differences between EcMglB and TpMglB-2 raise the question of whether their similarities are the result of convergent, rather than divergent, evolution. The preponderance of the evidence suggests that EcMglB and TpMglB-2 diverged from a common ancestor. The strong sequence conservation in the common parts of the proteins, including the conservation of a Ca^2+^-binding site, serves as ample evidence for this assertion. However, the poor sequence conservation between the permuted portions of the two proteins (and remembering that this part of TpMglB-2 includes a large insertion) may imply that TpMglB-2 and its close homologs acquired this part from a different gene, with evolutionary pressure influencing convergence of structural features.

### Functional Studies of TpMglB-2

**Fig 5:**
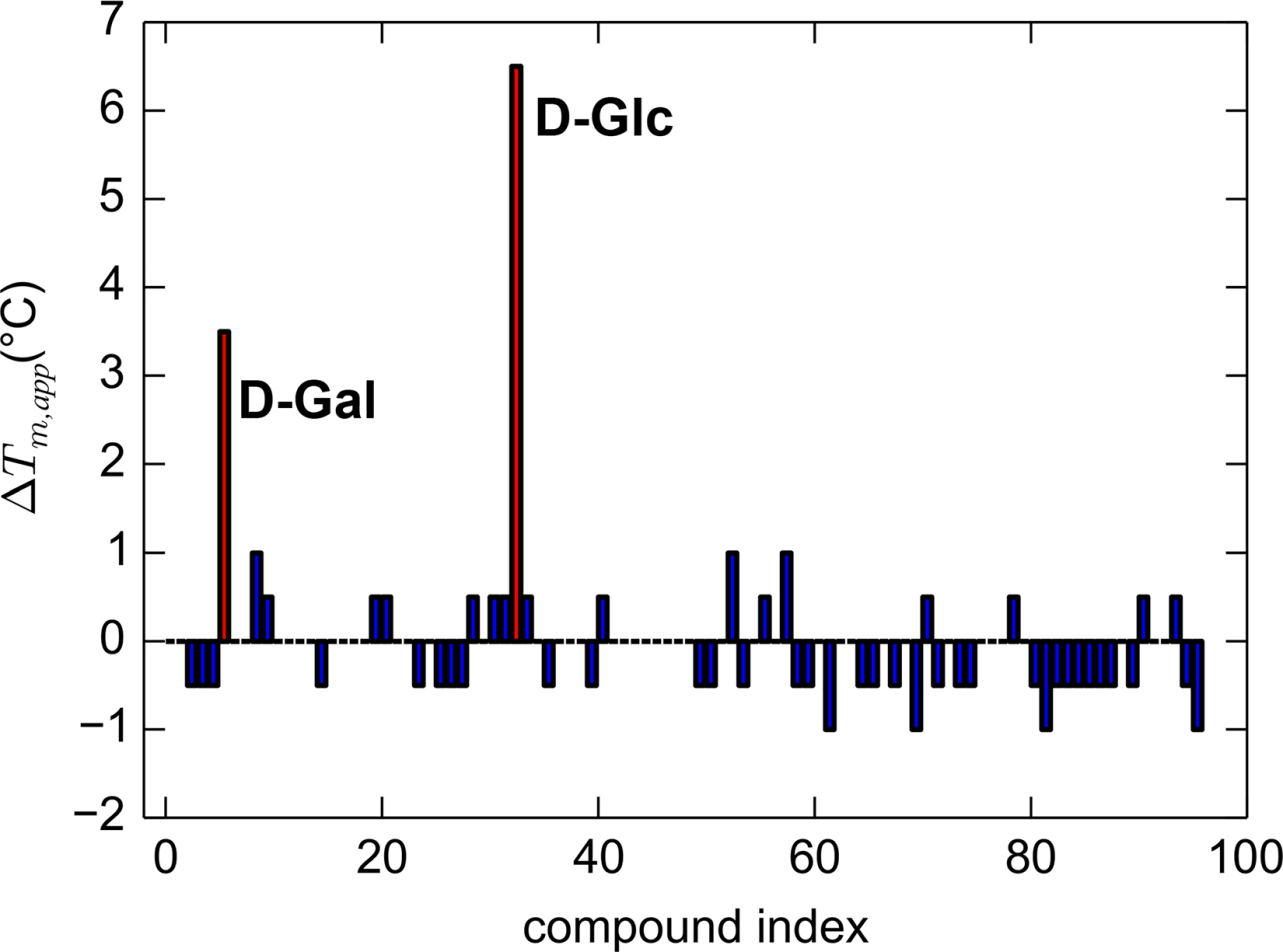
DSF data for a panel of carbon sources in the presence of TpMglB-2. The change in the apparent melting temperature is plotted vs. compound index (ranging from 1 to 96; only results from BIOLOG plate PM1 are shown here). Bars for compounds that caused *T*_*m*,*app*_ shifts of more than 1.5° C from that observed in the presence of the negative control (water, no compound) are colored red and labeled with the compound’s identity.

The unconventional structural organization and the altered Ca^2+^-binding site of TpMglB-2 provoked a question: does such a structurally rearranged LBP function similarly to other LBPs? To address this question, we first examined ligand binding. In a previous study on TpMglB-2, we reported that the protein preferred D-glucose and D-galactose over a small number of other monsaccharide carbon sources [37]. However, we wished to interrogate a larger panel of such sources to investigate whether another ligand would bind to the protein with even higher affinity. We thus employed differential scanning fluorimetry (DSF) as a means to probe for ligand binding [52,53]. This method examines the apparent melting temperature (*T*_m, app_) of the protein; the “apparent” nomenclature is added in recognition of the fact that the reversibility of the transition was not tested in these experiments. Presumably, in the presence of a specifically bound ligand, the protein’s structure will be stabilized, resulting in a higher *T*_m, app_. We used this plate-based method to qualitatively assay for the binding of 190 carbon sources. Only D-glucose and D-galactose caused significant shifts (> 1.5° C) in *T*_m, app_ (Fig 5). Thus, these were the only candidate compounds that were selected for additional study.

**Fig 6:**
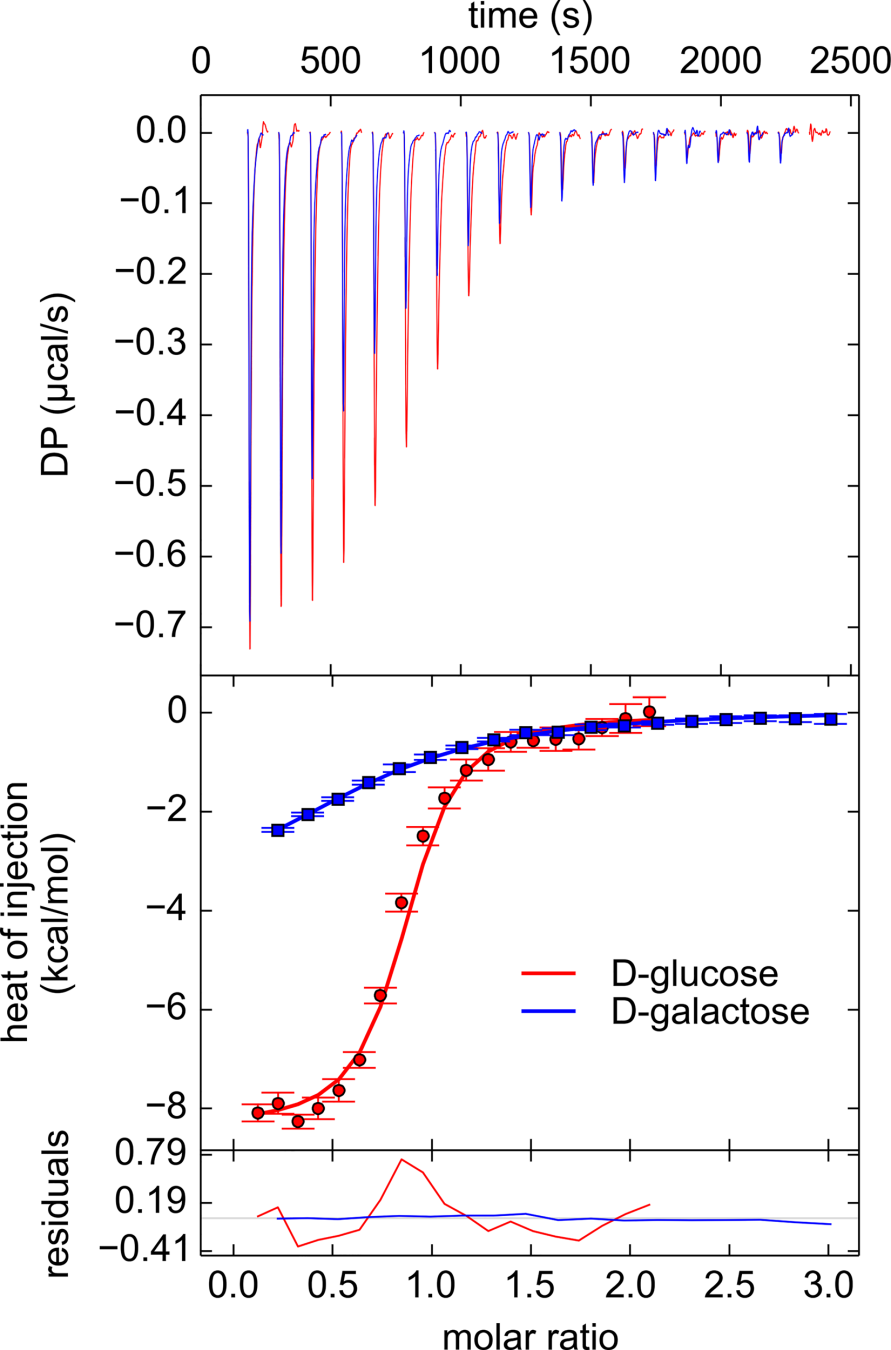
D-glucose and D-galactose binding TpMglB-2. Baseline-subtracted thermograms reconstructed from the raw data using singular-value decomposition are shown in the top panel. In the middle panel are the integrated heats of injection (markers) with error bars depicted estimated measurement errors [54,55]. The lines are fits to these data points. The bottom panel shows the residuals between the data and the fit lines. All lines and markers are colored respectively according to the inset legend. Only one of the three analyzed experiments is shown for each monosaccharide.

ITC was used to quantify the interactions between TpMglB-2 and the subject monosaccharides. Indeed, both of these sugars elicited robust heat signals when titrated into solutions of the protein (Fig 6). D-Mannose and D-ribose, which both apparently failed to bind in the DSF assay, were titrated in control experiments. Corroborating the DSF experiments, they failed to exhibit any binding heats.

Analysis of the binding isotherms resulting from these experiments shows that TpMglB-2 has a pronounced preference for D-glucose over D-galactose in this assay: the measured *K*_D_ for D-galactose was 30-fold higher than that of D-glucose (Fig 6; Table 3). Although the data were analyzed using a 1:1 binding model, it was always necessary to refine a parameter [55,56] that compensated for an apparent incompetent fraction of the protein (15–30%). The modest *K*_D_ for D-glucose binding to TpMglB-2 (1.1 μM) should not hamper import of the hexose, as its extracellular concentration in *T*. *pallidum*’s environments is expected to be in the millimolar range [36].

**Table 3:** Dissociation constants of the TpMglB-2/sugar interactions.

TpMglB-2 Variant	Sugar	*K*_D_ (μM)
wild-type	D-glucose	1.1 [0.9, 1.4]^a^
wild-type	D-galactose	33 [21, 58]
N19A	D-glucose	NH^b^
W145A	D-glucose	12.7 [10.6, 15.5]
D195A	D-glucose	NH
F313A	D-glucose	NH

^a^68.3% confidence intervals are given in brackets.

^b^NH: No observable heat of ligand binding.

These results contrast with those that we obtained earlier using a similar TpMglB-2 construct [37]. In those experiments, TpMglB-2 bound to both D-glucose and D-galactose with similar, sub-micromolar equilibrium dissociation constants (150 nM for D-glucose, 250 nM for D-galactose). Although the exact reason for this discrepancy is unknown, it is notable that the present experiments were carried out with fivefold higher NaCl concentrations (20 mM vs. 100 mM). The ionic strength of the present experiments is likely closer to that of the *T*. *pallidum* periplasm, rendering the measurements presented here more physiologically relevant.

**Fig 7:**
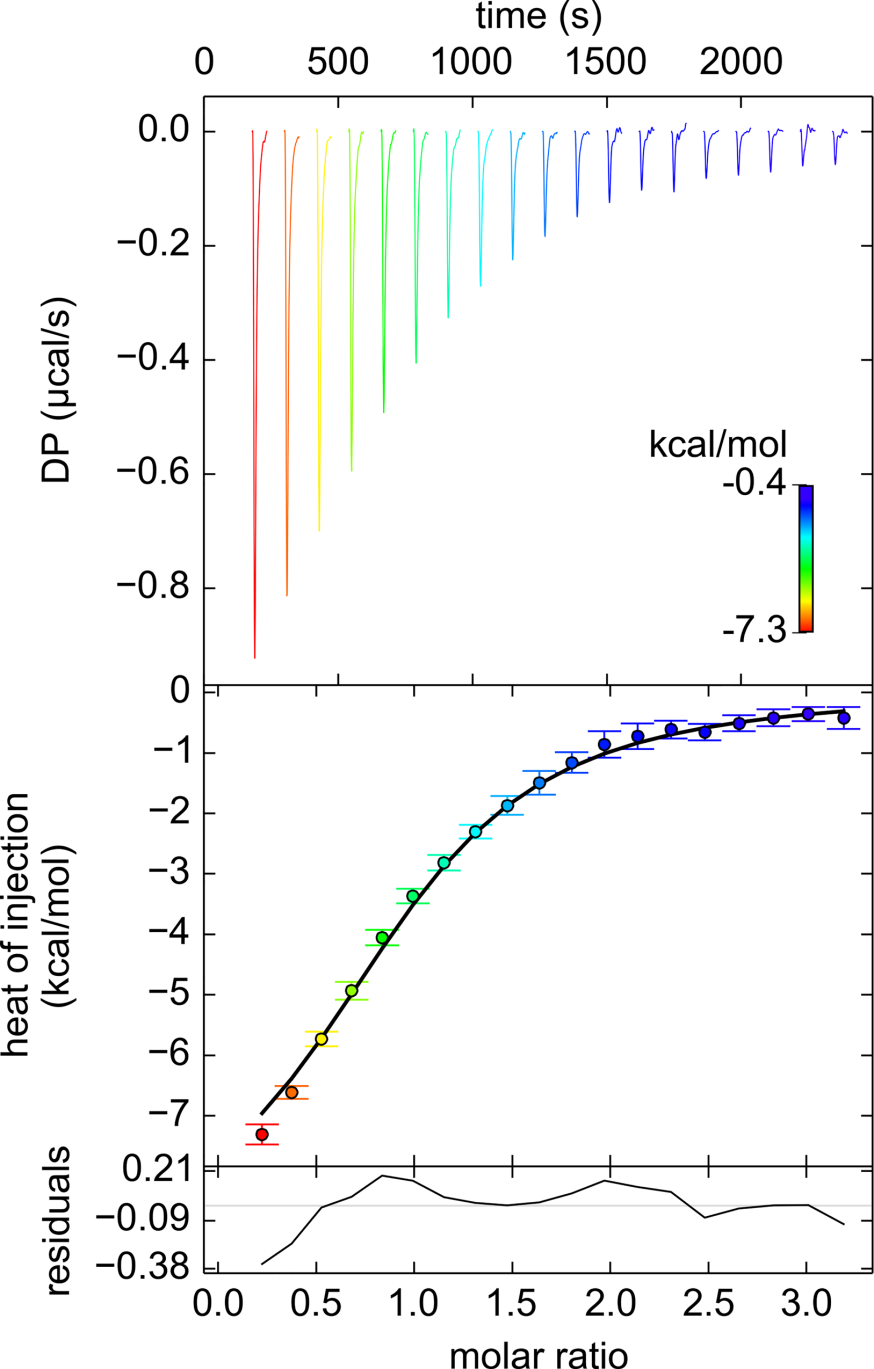
D-glucose binding to the W145A variant of TpMglB-2. The organization of the figure is the same as in Fig 6, but the thermogram peaks and their respective integrated data points are colored according to the color map shown inset in the top panel.

To determine whether the putative binding site—identified by the binding site of BIS-TRIS (Figs 1 & 2) and by homology to the EcMglB binding site (Table 2)—is responsible for binding D-glucose in TpMglB-2, we engineered single-amino-acid changes into the protein and assessed the affinity of the mutant proteins for the hexose (Table 3). None of the mutant proteins (except W145A) displayed an interpretable heat signal for D-glucose binding, strongly suggesting that their D-glucose-binding functions were severely diminished. Analytical ultracentrifugation (AUC) in the sedimentation velocity (SV) mode was performed on the wild-type protein and all of the mutated proteins. All had identical hydrodynamic radii, suggesting that the mutations did not cause large-scale changes in the overall structures of the proteins. The one mutant protein that did display binding heats, W145A, had a tenfold higher *K*_D_ for glucose binding than the wild-type protein (Table 3; Fig 7). From these data, we conclude that binding site identified above is very likely to be correct.

The hydrodynamic characteristics of TpMglB-2 gleaned from SV were further scrutinized with respect to the protein’s oligomeric state. We found that this protein was monomeric under our experimental conditions at all concentrations studied (0.6–5 mg/mL) and in the presence and absence of D-glucose. While these data comport with data from other glucose-binding LBPs [49,57], they contrast with reports on other LBPs that can form dimers [58,59].

We also wished to discover whether the topologically distinct TpBglB-2 undergoes a conformational change upon ligand biding; such conformational changes have been observed in other D-glucose-binding LBPs in the crystalline state [48] and in solution [49,57], consistent with the “Venus fly trap” model. All attempts to co-crystallize D-glucose with TpMglB-2 or to introduce it into the crystals described above failed; we do not believe that this failure is due to the circular permutation in the protein disrupting its ability to co-crystallize. Nonetheless, we again chose to characterize the hydrodynamic traits of the protein in the presence and absence of D-glucose. The “closed” forms of LBPs should exhibit a smaller hydrodynamic radius (i.e. larger sedimentation coefficient) because of their more compact conformation. Careful hydrodynamic modeling of liganded (2FVY) and unliganded (2FW0) crystal structures indicated that the TpMglB-2 structural homolog, EcMglB, confirmed this assertion; the protein would evince a 0.080-S higher sedimentation coefficient upon ligand binding. While this change seems quite small (i.e. a 2.5% change in *s*-value), discerning such differences is well within the capabilities of modern instrumentation and analytical methods [60,61].

Our initial attempt to examine whether this small difference could be detected was conducted using conventional SV. In experiments carried out simultaneously, we sedimented apo-TpMglB-2 and also the protein in the presence of 100 μM D-glucose. By analyzing the data with a non-interacting discrete species model [62], we could perform a careful analysis using F-statistics [63] that would establish realistic 95% confidence intervals (shown below in brackets) on the refined parameter *s*. For apo-TpMglB-2, the refined experimental *s*-value was 3.212 [3.204–3.219] S. The same value of the protein in the presence of D-glucose was 3.292 [3.288–3.297] S. We can estimate the statistical significance of this difference the F-statistic formalism. If we fix the *s*-value of the apo-protein to that of the liganded protein and repeat the analysis, we arrive at a *χ*^2^ ratio between the two analyses of 1.86. Given the degrees of freedom in this experiment, it is extremely improbable (probability ≈ 10^−16^) that there is no difference in the *s*-values.

**Fig 8:**
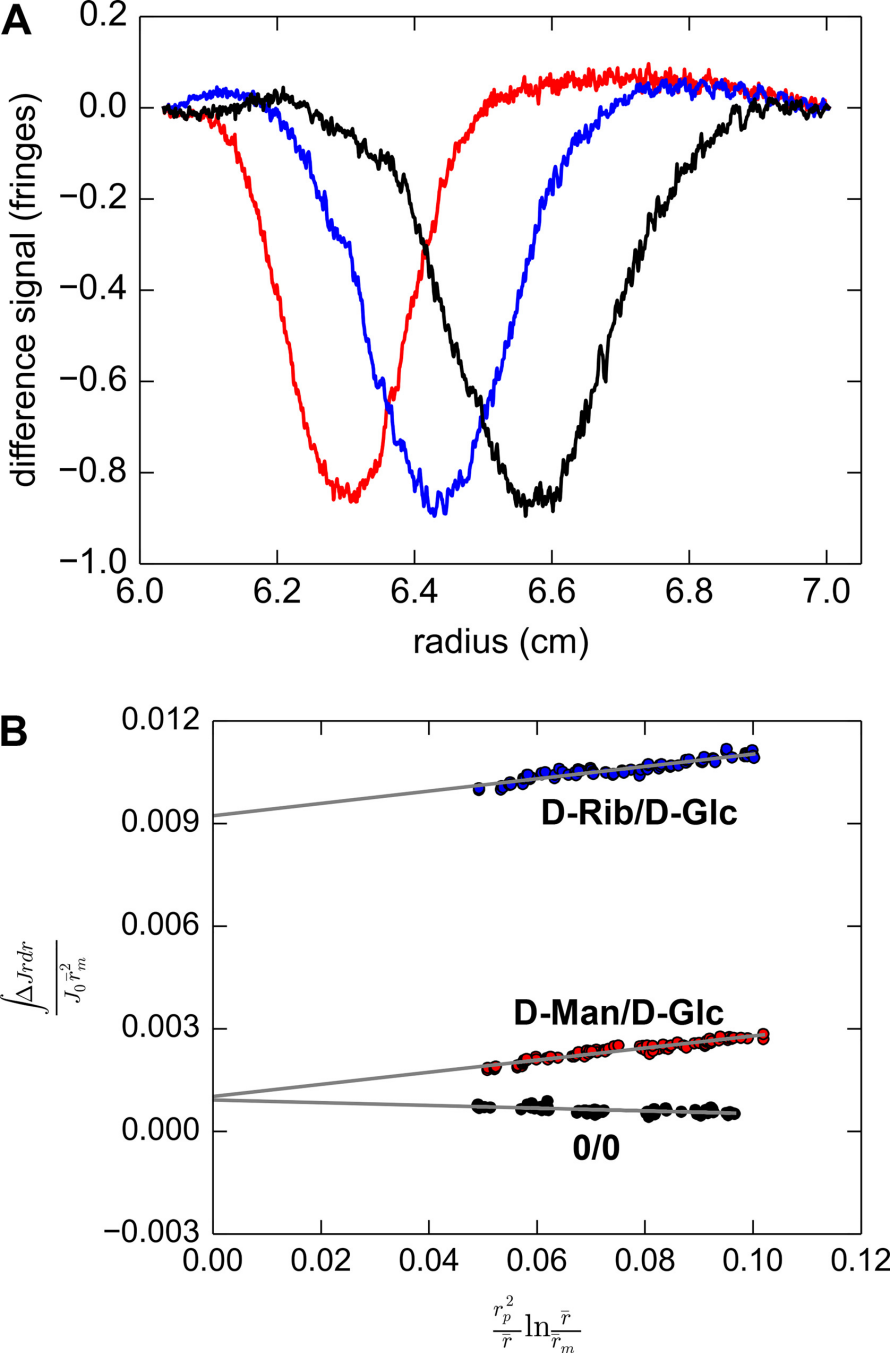
Difference sedimentation velocity of Tp0684 in the presence and absence of sugars. (A) Example difference SV traces. Three traces are shown after noise and baseline subtraction. The traces are colored according to the time of the scan, with red, blue, and black representing 92, 132, and 172 min after the start of centrifugation. The three traces are from the D-mannose/D-glucose experiment. (B) Three difference sedimentation velocity analysis plots. Data similar to those shown in (A) were treated as described in Materials and Methods; each trace resulted in one of the data points shown here, which are colored according to their respective experiments. Each experiment is labeled; “D-Man/D-Glc” stands for D-mannose vs. D-glucose, “D-Rib/D-Glc” is the D-ribose vs. D-glucose experiment, and “0/0” is the negative control in which no sugar was added to either sector. The gray lines show the linear fits to each data set. Eighty-six traces were analyzed per experiment. The D-Rib/D-Glc data are displaced upward because of the larger difference in the reference/sample sector menisci in this experiment (see Materials & Methods).

Although the previous SV study strongly suggested a conformational change, we sought a more direct comparison of the two sedimentation rates. Minute sedimentation-coefficient differences are readily detected using a method pioneered in H. Schachman’s lab called “difference sedimentation velocity” [64]. This method provides a direct, side-by-side comparison of the sedimentation velocity of two macromolecular solutes. In brief, two samples of 5 mg/mL TpMglB-2 were prepared. To one sample, a non-binding sugar, either D-mannose or D-ribose (these sugars evidenced no binding in neither the DSF nor ITC assays), was introduced, whereas an identical concentration of D-glucose was added to the other. The protein sample with the non-binding sugar was placed in the reference sector of a centrifugation centerpiece, and the sample sector of the same centerpiece was filled with the D-glucose-containing protein solution. Centrifugation was initiated, and Rayleigh laser interferometry was used to monitor the sedimentation. Because this mode of data collection reports on refractive-index differences between the two sectors, the difference between the sedimentation in the two sectors is recorded. This signal (for unequal solution-column heights) will appear roughly as a Gaussian-shaped curve inverted about the radial axis (Fig 8A). If the macromolecular solutes in each sector sediment at different rates, the absolute value of the area of (or, more specifically, the normalized first moment of) this curve will increase as sedimentation continues. This trend reports on the fractional sedimentation-coefficient difference between the solutes in the reference and sample sectors, termed $\Delta s/\bar{s}$, i.e. the change in *s* normalized by the mean *s*-value. Thus, when a normalized first moment of the interference signal is plotted as shown in Fig 8B, the slope of the fitted line is taken as $\Delta s/\bar{s}$. A nonzero slope is considered to be strong evidence of a sedimentation coefficient difference between the solutes in the two sectors.

As shown in Fig 8B, we observed a $\Delta s/\bar{s}$ of about 0.0177 [0.0163, 0.0191], or about 1.8% (for $\Delta s/\bar{s}$ values, we present the 95% confidence interval for the fitting in brackets) when comparing the sedimentation of TpMglB-2 in the presence of D-mannose/D-glucose. When repeating the experiment using the D-ribose/D-glucose pair, the resulting $\Delta s/\bar{s}$ was nearly identical (0.0180 [0.0163, 0.0197]). Multiplying $\Delta s/\bar{s}$ by the observed *s* for this same concentration of TpMglB-2, 3.2 S, results in an observed change of approximately 0.06 S. This correlates well with the difference obtained from comparing the *s*-value of individual experiments and also with the hydrodynamic modeling performed on the structural homolog EcMglB (see above).

An important control experiment in difference SV is to conduct the study with identically prepared solutions in each sector. When applied to TpMglB-2, this approach resulted in a weak negative trend in the first moments, eliciting a $\Delta s/\bar{s}$ of -0.0040 [-0.0052, -0.0028]. The fact that this is nonzero may indicate that this is the inherent error in the experiment or that there is a systematic -0.4% offset in the values obtained using our instrument. A systematic study of the errors associated with this method is required to distinguish between these possibilities.

Another critical consideration in difference SV experiments is the $\Delta s/\bar{s}$ expected solely from the gain in mass resulting from ligand binding. We calculated this (see Materials & Methods) to be 0.006, or about 0.6%. This value is well outside the 95% confidence intervals for our glucose-containing experiments. This result, coupled with the close correlation between the solution experiments and hydrodynamic modeling, allowed us to conclude that it is very likely that a conformational change occurs when D-glucose binds to apo-TpMglB-2.

## Discussion

*T*. *pallidum* is thought to be microaerophilic[65], and there is no evidence that the electron-transport chain that would allow canonical aerobic respiration exists in the organism [30]. Further, D-glucose is believed to be the main, if not sole, carbon source for the microorganism [32–35]. Thus, a modified glycolytic pathway could be the only means for energy generation in *T*. *pallidum*. These facts make the study of the cellular import of D-glucose critical for understanding the lifestyle of the organism. In this report, we found that TpMglB-2, the product of gene *tp0684*, is very likely the D-glucose-binding protein of an ABC transporter dedicated to the import of this vital molecule. This conclusion identifies this protein as a crucial cog in the machinery of energy acquisition for *T*. *pallidum*. TpMglB-2 has an unconventional topology for an LBP (Fig 3), but retains the hallmarks of ligand binding and ligand-induced conformational change that are common to this family of proteins (Figs 5–8). Further, its marked preference for D-glucose over D-galactose is consistent with the likely paucity of the latter sugar in native (human) *T*. *pallidum* environments.

The unusual topology (Figs 1–3) of TpMglB-2 is present in putative sugar-binding proteins in other bacteria (Fig 4). The structure detailed herein thus serves as the founding member of a family of proteins related to the “Type I” [39] or “Cluster B” [40] LBPs. We propose that these proteins belong to a sub-class/cluster of these classification systems, given the strong structural homology to canonical members. We suggest that they TpMglB-2-like proteins be referred to as “Type I_c_” or “Cluster B_c_”, with the subscripted “c” appended to note the circular permutation of this subclass.

TpMglB-2 homologs (in both sequence and topology) are found mostly in other treponemes or Gram-positive bacteria. A noteworthy commonality among most of these organisms is that their respective LBPs are probably tethered. In the treponemes, these proteins are membrane-tethered in the periplasmic space, and they are probably attached to the outer cell wall in Gram-positive bacteria. These facts raise the intriguing possibility that this divergent topology arose as an adaptation to the necessity of anchoring the proteins to a membrane. No physical or mechanical advantage to this topology is immediately obvious from the structure, but we do note that the N-terminus of EcMglB is near to two sugar-binding amino acids (D14 and F16), whereas the topologically distinct N-terminus of TpMglB-2 is near to only one (N19). Therefore, strain on the N-terminus induced by tethering and Brownian motion could have a more adverse effect on the binding of D-glucose in the *E*. *coli* protein. Another possibility is that the different topology better satisfies the requirements of the export/lipidation machinery in these organisms.

An important question to resolve regarding the biology of *T*. *pallidum* is whether TpMglB-2 and its cognate ABC permease/ATPase represent the organism’s only means of glucose uptake. Another protein, the product of *tp0545*, also has sequence homology to EcMglB and has been termed “TpMglB-1” [30,37]. The *tp0545* gene does not appear to be in an operon with other ABC transporter components. Hidden-Markov-model searches [45] of the Protein Data Bank reveal a high probability that TpMglB-1 is structurally similar to EcMglB. Efforts to produce a recombinant form of TpMglB-1 to test its sugar-binding capabilities have thus far failed. However, a sequence alignment of this protein with EcMglB and TpMglB-2 demonstrates that certain sugar-binding residues are not conserved (Table 2). This result casts doubt on the ability of TpMglB-1 to serve as a D-glucose-binding protein. Thus, TpMglB-1 may resemble EcMglB only insofar as its structural homology to that protein.

As detailed elsewhere [38], the *E*. *coli* structural homolog of TpMglB-2, EcMglB, is a multifunctional protein. In addition to its role as the LBP for a D-glucose/D-galactose ABC transporter, it is also the chemoreceptor for a well-studied sugar chemotaxis system. We and others [37,66,67] have noted that all of the essential elements of a homologous chemotaxis apparatus are apparently present in *T*. *pallidum*. Indeed, motility and chemotaxis are likely major mechanisms for this spirochete to invade tissues distal from the site of initial infection [68,69], thus clinically manifesting as secondary and tertiary syphilis. If TpMglB-2 is also a chemoreceptor for a D-glucose-tactic system, its proper functioning has implications not only for spirochetal survival but also for pathogenicity.

## Conclusions

TpMglB-2 adopts a fold that is common for periplasmic LBPs; however, it displays a hitherto unknown topology for this class of proteins. Careful investigation of the binding and physical characteristics of the protein are consistent with the notion that this protein is acting as the ligand-binding element of an ABC transporter for D-glucose. This assertion is buttressed by noting that the gene for this protein, *tp0684*, is clustered with probable transmembrane and ATP-binding elements of the cognate ABC transporter. As no other carbon source has been reliably identified that supports the growth and chemotaxis of *T*. *pallidum*, this transporter has implications for the survival and pathogenicity of this bacterium.

## Materials & Methods

### Cloning, overexpression, and protein preparation

To produce a non-lipidated, recombinant derivative of TP0684 in *E*. *coli*, the DNA fragment encoding amino acid residues 11–378 (cloned without the post-translationally modified N-terminal Cys plus nine other residues comprising the predicted N-terminal transmembrane helix) of TP0684 was PCR amplified from *T*. *pallidum* genomic DNA by the polymerase incomplete primer extension (PIPE) cloning method using ends-specific primers (PIPE insert). The expression vector, pSpeedET (DNASU, Tempe, AZ), which encodes an N-terminal TEV-protease cleavable expression and purification hexa-histidine tag (MGSDKIHHHHHHENLYFQG), was PCR amplified with PIPE-vector primers. The PIPE-insert and PIPE-vector was mixed to anneal the amplified DNA fragments together[70]. *E*. *coli* HK100 competent cells were transformed with the mixtures (PIPE-vector and insert) and selected for kanamycin resistance on LB agar plates. Cloning junctions/fragments were verified by DNA sequencing. A verified plasmid was then co-transformed with pGroESL (Takara, Shiga, Japan) into *E*. *coli* BL21 AI (Invitrogen) cells for soluble protein expression. *E*. *coli* BL21 AI cells were grown at 37° C in LB medium containing 0.1% (w/v) glucose, 40 μg/mL of kanamycin and 30 μg/mL of chloramphenicol until the cell density reached an A600 of 0.5. The culture was then induced for overnight at 16° C with 0.2% (w/v) L-arabinose. The procedures for expression and purification of the recombinant proteins were essentially as previously described [22].

For the production of selenomethionine labeled protein, *tp0684* was recloned into a pProEx HTb vector (Invitrogen) and co-transformed with pGroESL (Takara) into a methionine auxotroph *E*. *coli* B834 (DE3). The recombinant protein was overproduced and purified as described previously [24].

### Site-directed mutagenesis and protein concentration determination

For the construction of the recombinant TpMglB-2 variants, the N19A, W145A, D195A and F313A mutation was individually introduced into the plasmid carrying the wild-type *tp0684* sequence using the QuikChange site-directed mutagenesis kit (Agilent Technologies, Santa Clara, CA). The mutation was confirmed by DNA sequencing. The mutant protein was expressed and purified as described above. Protein concentrations were determined in buffer A (20 mM Hepes, 0.1 M NaCl, pH 7.5, 2 mM n-Octyl-β-D-glucopyranoside) using spectrophotometry. Extinction coefficients were calculated using the Protparam tool of ExPASy (www.expasy.org).

### Crystallization and cryoprotection

Crystals of TpMglB-2 were obtained by mixing 4 μL of TP0684 (~22 mg/mL in buffer A) with 4 μL of crystallization buffer (0.1 M BIS-TRIS, pH 6.5, 20% (w/v) PEG MME 5,000) and incubating them over 0.5 mL of the reservoir (containing crystallization buffer) for 10 days. The crystals were transferred to the stabilization buffer (SB: 0.1 M BIS-TRIS, pH 6.5, 100 mM NaCl, 20% (w/v) PEG MME 5,000, 5% (v/v) ethylene glycol). After about 5 minutes in SB, they were serially transferred to buffers that were the same as SB except that they had higher concentrations of ethylene glycol. The final concentration of ethylene glycol was 25%. After about 1 min in this solution, the crystals were flash-cooled in liquid nitrogen. Toward solving the phase problem, crystals of a selenomethionyl derivative of TpMglB-2 (SeTpMglB-2) were grown by mixing 4 μL of SeTpMglB-2 (~9 mg/mL in buffer A) with 4 μL of crystallization buffer (0.1 M BIS-TRIS, pH 5.5, 0.1 M ammonium acetate, 17% (w/v) PEG 10,000) and incubating them over 0.5 mL of reservoir solution containing the crystallization buffer for 7 days. The crystals were transferred to the stabilization buffer 2 (SB2: 0.1 M BIS-TRIS, pH 5.5, 0.1 M ammonium acetate, 17% (w/v) PEG 10,000, 5% (v/v) ethylene glycol) and cryoprotected/flash cooled as above.

### Data collection, structure determination and refinement

All X-ray diffraction data were acquired at beamline 19-ID of the Structural Biology Center at Argonne National Laboratories. Native TpMglB-2 crystals had the symmetry of space group C222_1_ and diffracted X-rays with a *d*_min_ spacing of 2.05Å. The data were reduced and scaled using HKL3000 [71]. Phase determination was undertaken using single-wavelength anomalous diffraction from crystals of a SeTpMglB-2 (see above). These crystals had the same symmetry as the native crystals and diffracted with a similar *d*_min_ spacing, but exhibited significantly divergent unit-cell constants. Diffraction data from the selenomethionine-derivatized crystals were reduced and scaled using HKL3000. The GUI interface in that program was utilized to perform a substructure solution (using SHELXD [72]), site refinement (using MLPHARE [73]), density modification (using DM [74]; no non-crystallographic symmetry is present), and automated model building (using Arp [75]). This strategy resulted in a model that was 93% complete. This model was used as a molecular replacement model to determine phases for the native structure using Phaser[76]. The model was completed and corrected in Coot [77]. After an initial round of simulated annealing, the model was refined in PHENIX [43] using positional and TLS refinement. Riding hydrogen atoms were included in the model. The weights between the chemical and X-ray terns were refined to optimize *R*_*free*._ The final model had excellent statistics and geometry (Table 1), and it featured one molecule of BIS-TRIS and one Ca^2+^ cation. The model and structure factors for TpMglB-2 have been deposited in the Protein Data Bank with accession number 5JX2. Structure figures were rendered using PyMol (Schrödinger LLC).

### Isothermal titration calorimetry

TpMglB-2 was dialyzed exhaustively against Assay Buffer (10 mM sodium phosphate, pH 7.4, 100 mM NaCl). The sugars to be titrated were dissolved in the same buffer. All titrations were carried out in an iTC200 calorimeter (Malvern, Malvern, UK). A typical titration scheme featured 19 or 20 2-μL injections of 500 μM of the monosaccharide into 50 μM protein in the ca. 200-μL stirred interaction cell. However, the concentrations were sometimes altered to sample a wider range of concentration space[56]. Three titrations were performed per sugar, and the resulting thermograms were serially integrated using NITPIC v. 1.1.7 [54,78]. All titrations for a given sugar were globally analyzed in ITCsy[55], and confidence intervals were obtained using the error-surface projection method[55,63]. All ITC illustrations were rendered in GUSSI [79].

### Hydrodynamic modeling

HYDROPRO [80] was used to carry out all hydrodynamic modeling in this study. For apo-EcMglB, the molar mass (*M*) and partial-specific volume ($\bar{v}$) were calculated using SEDNTERP [81] and inputted into HYDROPRO. The crystal structure used was 2FW0 [48] denuded of all water molecules and ligands (although this structure is described as “apo”, there is a citrate molecule located in the D-glucose-binding site). For modeling of the liganded, “closed” structure, 2FVY [48] was used. The procedure was the same, but mass (180 g/mol) was added and the weighted-average $\bar{v}$ was calculated to account for the bound ligand.

### Analytical ultracentrifugation

All AUC experiments were carried out at 20° C in a Beckman-Coulter Optima XL-I centrifuge (Beckman-Coulter Inc., Indianapolis, IN), and Assay Buffer was used in all cases. Charcoal-filled Epon centerpieces with 1.2-cm path-lengths were housed between two sapphire windows in a standard aluminum housing. After introduction to the centerpiece sectors, all sample cells were placed in an An50-Ti rotor that was subsequently incubated at the experimental temperature under vacuum in the centrifuge for at least 2.5 h prior to the initiation of rotation. The rotor speed was 50,000 rpm for all AUC studies. For normal SV experiments, 400 μL of Assay Buffer was introduced into the reference sector, and 400 μL of sample was placed in the sample sector. Centrifugation continued for about 16 h, and concentration-profile data were collected using both absorbance and interference optics. Data were initially analyzed using the *c*(*s*) method in SEDFIT [82,83]. As warranted, data were also analyzed using a discrete-species model in SEDPHAT [62].

For difference SV studies, a slightly different protocol was followed. First, a 5 mg/mL stock of TpMglB-2 (in Assay Buffer) was made, and stock solutions of the sugars to be examined were also prepared in Assay Buffer. Identical volumes of the sugars were dispensed to separate tubes, followed by the addition of identical volumes of the protein stock solution. The two pairs of sugars studied were D-mannose/D-glucose and D-ribose/D-glucose. In a third experiment (a negative control), no sugars were added, only Assay Buffer. The solution containing the non-binding sugar was placed into the reference sector of a centerpiece, and the solution containing D-glucose was inserted in the sample sector. For the D-mannose/D-glucose and buffer/buffer experiments (performed on different days), the experiment was carried out in a meniscus-matching centerpiece (Spin Analytical, Inc., Berwick, ME). Thus, ca. 10 μL less of the glucose containing solution was dispensed than that of the reference-sector solution. The solution-column heights were equalized prior to the experiment by 30 min of centrifugation at 9,000 rpm. After equalization, the centrifugation cell was removed and repeatedly inverted to thoroughly mix the sector contents. The equalization step resulted in a small amount of non-binding sugar being transferred to the sample sector. The D-ribose/D-glucose experiments were done in a normal Beckman-Coulter centerpiece, so the column-height equalization step was not possible. A deliberate meniscus mismatch was introduced into this experiment by pipetting ca. 6 μL less into the sample sector compared to the reference sector, as suggested [64]. The same pre-experiment incubation period and rotor speed as described above were utilized for these experiments. A “fringe-control” experiment in which buffer was placed in the reference sector and an identical concentration of protein (compared to the difference SV experiments) was always run side-by-side with the difference SV experiments. Sedimentation and sedimentation differences were monitored using the interference optics only; scans were obtained every 60 s for almost 17 h.

As presented by the data-acquisition software, interference data have the amount of fringe displacement, *J*, on the y-axis and the radius from the center of rotation on x-axis (*r*). In this case, of course, the difference in fringe displacement, Δ*J*, was measured. Difference SV data, as mentioned above, take on the appearance of a Gaussian distribution inverted about the x-axis (Fig 8A). The details of the analytical method will be published elsewhere (C.A.B., R.K.D., & M.V.N., in preparation). Briefly, to analyze these data, we first identified by eye which scans displayed a suitable amount of both meniscus depletion and evident plateau regions. Usually a subset of ca. 80 scans was suitable for analysis under this criterion. Next, we determined the time-invariant noise in the scans by examining the last 10 scans (in which no sedimentation was occurring, as solute had been depleted from the analysis range) and calculating the noise assuming no sedimenting species in SEDFIT [84]. This noise was subtracted from the subject difference-data scans. Next, the first moment of the difference curve by first subtracting a baseline value and then finding the area above the curve Δ*J*⋅*r*, in essence performing the integral

$$\int_{r_{1}}^{r_{2}}\Delta Jrdr,$$ (1)

where *r*_1_ and *r*_2_ are dynamically chosen radial values proximal to the meniscus and plateau regions, respectively. These values were normalized by quantity $J_{0}{\bar{r}}_{m}^{2}$, where *J*_0_ is the total amount of fringe displacement of the protein (from the “fringe-control” experiment described above) and ${\bar{r}}_{m}$ is the average of the two menisci in the difference SV experiment. Thus the quantity

$$\left|\frac{\int_{r_{1}}^{r_{2}}\Delta Jrdr}{J_{0}{\bar{r}}_{m}^{2}}\right|$$ (2)

was tabulated for every scan. Finally these data were plotted as the ordinate for the respective abscissa

$$\frac{r_{2}^{2}}{\bar{r}}\ln\frac{\bar{r}}{{\bar{r}}_{m}},$$ (3)

where $\bar{r}$ is the radial position of the minimum in the Δ*J* curve. These data were fitted to a straight line, and the slope was taken as the quantity $\Delta s/\bar{s}$. The 95% confidence interval of this value was determined using F-statistics and the error-surface projection method, as discussed elsewhere[63]. Estimation of $\Delta s/\bar{s}$ for the addition of ligand without conformational change was accomplished using Eq. 19 of Kirschner & Schachman [64].

### Differential scanning fluorimetry

The thermal stability of TpMglB-2 in the presence of various putative ligands was determined in a 96-well PCR-plate (Bio-Rad Laboratories, Inc., Hercules, CA). The assumption was that binding ligands would enhance the thermal stability of the protein. For carbon-source screening, Biolog's Phenotype MicroArray (PM) compounds supplied in 96-well microplates (BIOLOG, Inc., Hayward, CA) were dissolved in 50 μL of sterile water to obtain a final concentration of around 10–20 mM. Screenings were performed with plates PM1 and PM2A. Each plate contains 95 compounds and a blank (no ligand) control. The complete plate contents are available at the BIOLOG website (www.biolog.com).

Each 20 μL standard assay mixture in a 96-well PCR-plate contained 10 μM purified protein and SYPRO Orange (Life Technologies) at 5x concentration in a buffer containing 10 mM phosphate, 100 mM NaCl, pH 7.4. Two μL of the resuspended BIOLOG compounds were added to each well. The plate was sealed and placed in a BioRad CFX96 real-time PCR detector coupled to a C1000 thermal cycler (BioRad Laboratories, Inc., Hercules, CA), and the fluorescence of the dye was monitored as a function of temperature from 4° C to 95° C. Fluorescence readings were recorded every 0.5° C. The data were transformed to–dF/dT v. T curves, and the abscissas of the minima in these curves were defined as the apparent melting temperatures (*T*_*m*, *app*_). Significant positive shifts in *T*_*m*, *app*_ were taken as positive results and were confirmed using ITC.

### Bioinformatics

BLAST [51] was used to identify protein sequences similar TpMglB-2. All hits were visually examined to ensure that full coverage of the TpMglB-2 was achieved. The top 100 hits were assembled, and identical or very similar sequences (i.e. they contained identical sequential cores with differences only on the periphery) were eliminated. In the end, 78 sequences were analyzed, including the outgroup of two sequences (MglB proteins from *E*. *coli* and *S*. *typhimurium*). PROMALS-3D [85] was used to align the sequences using the current structure of TpMglB-2 as a structural template. This output was converted to Phylip format using the online resource at insilico.ehu.es/tophylip/. PhyML[86] was applied to establish the maximum-likelihood tree for the protein sequences. The output from this session was visualized by running a short Python script that utilized the toolkit ETE [87] version 3.

## Supporting Information

S1 File Raw Data Presented in this Paper. File S1_File.7z is a compressed folder containing the raw data for the AUC, ITC, and bioinformatics results described in this paper.
(7Z) Click here for additional data file.

## Abbreviations

3-OMe Glc: 3-*O*-methyl glucose
AUC: analytical ultracentrifugation
DSF: differential scanning calorimetry
EcMglB: the MglB protein from *E*. *coli*
ITC: isothermal titration calorimetry
LBP: ligand-binding protein
r.m.s.d.: root-mean-squared deviations
SV: sedimentation velocity
TpMglB-2: the seconds MglB homolog from *T*. *pallidum*
